# Added Value of Arterial Enhancement Fraction Color Maps for the Characterization of Small Hepatic Low-Attenuating Lesions in Patients with Colorectal Cancer

**DOI:** 10.1371/journal.pone.0114819

**Published:** 2015-02-23

**Authors:** Mina Park, Yong Eun Chung, Kyung Ah Kim, Woo-Suk Chung, Hye Sun Lee, Kyung Hwa Han, Myeong-Jin Kim, Ki Whang Kim

**Affiliations:** 1 Department of Radiology, Research Institute of Radiological Science, Yonsei University, College of Medicine, Seoul, Korea; 2 Department of Radiology, St. Vincent’s Hospital, The Catholic University of Korea, Suwon, Korea; 3 Department of Radiology, Konyang University College of Medicine, Daejon, Korea; 4 Biostatistics Collaboration Unit, Medical Research Center, Yonsei University College of Medicine, Seoul, Korea; Penn State Hershey Cancer Institute, UNITED STATES

## Abstract

**Objective:**

To assess the added value of arterial enhancement fraction (AEF) color maps for the differentiation of small metastases from hepatic benign lesions.

**Subjects and Methods:**

We retrospectively analyzed 46 patients with colorectal cancer who underwent multiphasic liver CT imaging and had low-attenuating liver lesions smaller than 3 cm (123 total lesions; metastasis: benign = 32:91). AEF color maps of the liver were created from multiphasic liver CT images using dedicated software. Two radiologists independently reviewed multiphasic CT image sets alone and in combination with image sets with AEF color maps using a five-point scale. The additional diagnostic value of the color maps was assessed by means of receiver-operating characteristic (ROC) analysis.

**Results:**

The area under the ROC curve (Az) increased when multiphasic CT images were combined with AEF color map analysis as compared with evaluation based only on multiphasic CT images (from 0.698 to 0.897 for reader 1, and from 0.825 to 0.945 for reader 2; P < 0.001 and 0.002, respectively). The increase Az was especially significant for lesions less than 1 cm (from 0.702 to 0.888 for reader 1, and from 0.768 to 0.958 for reader 2; P = 0.001 and P = 0.001, respectively). The mean AEF of tumor-adjacent parenchyma (35.07 ± 27.2) was significantly higher than that of tumor-free liver parenchyma (27.3 ± 20.6) (P = 0.04).

**Conclusions:**

AEF color mapping can improve the diagnostic performance for small hepatic metastases from colorectal cancer and may allow for the elimination of additional examinations.

## Introduction

The liver is the most common site of metastasis from colorectal cancer. Accurate preoperative diagnosis of liver metastases is critical for planning treatment and predicting prognosis [1,2]. Computed tomography (CT) is the primary imaging modality for preoperative evaluation of patients with known or suspected colorectal cancer and can be used to accurately diagnose both the extent of primary colorectal cancer and liver metastasis. Small, low-attenuation lesions in the liver were detected in 13% to 25.5% of preoperative CT scans, although only 2.2% to 14.0% were confirmed as metastases [3,4]. It is often difficult to characterize such small hepatic lesions on CT images. Although gadolinium ethoxybenzyl diethylenetriamine pentaacetic acid (Gd-EOB-DTPA)-enhanced magnetic resonance image (MRI) and positron emission tomography (PET) can be helpful for the diagnosis of small metastases, [5–7] these imaging modalities require additional expense and time and are not available in some facilities or countries.

Arterial enhancement fraction (AEF) color mapping can reveal hemodynamic changes in liver parenchyma. The AEF is defined as the ratio of the attenuation increment during the arterial and portal venous phases compared with the unenhanced image (AEF = [(HU_A_—HU_U_)/(HU_P_—HU_U_)], where HU is the attenuation, A is the arterial phase, P is the portal venous phase, and U is the non-contrast image [8]. Hepatic hemodynamic changes have been shown to precede definite liver metastasis and higher enhancement of liver between 25 and 40 sec can suggest occult liver metastasis [9,10]. AEF color mapping is also helpful for predicting tumor response to chemotherapy [11], and could improve the diagnostic performance for focal liver lesions such as HCC [8].

We hypothesized that semi-quantitative evaluation of small liver lesions and quantitative comparison of lesion-adjacent and lesion-free liver parenchyma with AEF color mapping may also improve the diagnostic performance for differentiating small liver metastases from hepatic cysts. The major advantage of AEF color mapping is that it is a pure post-processing technique, and could therefore be carried out using routine dynamic CT data without additional radiation or other imaging studies. The purpose of this study was to investigate the diagnostic value of the AEF for the differentiation of small hepatic metastases from benign hepatic cysts in patients with colorectal cancer.

## Materials and Methods

### Patients

We obtained approval for this retrospective study from the Institutional Review Board, and the requirement for informed consent was waived. We retrospectively reviewed electronic medical records and radiologic reports from between April 2009 and May 2011. Patients who had colorectal cancer and who underwent multiphasic CT including precontrast, arterial phase, and portal venous phase were included in the study. Patients who had hepatic metastases larger than 30 mm were excluded, because large liver metastases can easily be diagnosed as metastases, whereas the aim of our study was to evaluate the added value of AEF for detecting small hepatic metastases. Patients who underwent thermal ablation therapy for hepatic lesions were excluded as well to avoid misdiagnoses of standard references. Patients who had more than eight liver metastases or who had a history of any prior treatment for liver metastasis were also excluded from the study. In addition to histopathologic confirmation, all patients had undergone follow-up contrast-enhanced CT or MRI. Determination of metastasis was based on either histopathology or imaging surveillance. Without histopathologic reports, liver metastasis was confirmed when: 1) the lesion showed typical metastatic imaging findings [12–14]; and 2) interval size change was seen on serial imaging. Cysts were diagnosed based on typical imaging findings such as bright hyper-intensity on T2-weighted images and no enhancement on dynamic contrast-enhanced T1-weighted images or were diagnosed as such being water-attenuating lesions without growth in at least 12 months of follow-up on MDCT, MRI, or a combination of MDCT and MRI.

### CT Acquisition

CT examinations were performed using multidetector CT scanners (Somatom Sensation 16, Sensation 64, or Somatom Definition Flash; Siemens Medical Solutions, Forchheim, Germany). The scanning parameters were as follows: 120-kV; 240 reference mAs, slice thickness, 3 mm, and table speed of 18.64–26.75 mm/rotation (pitch 0.6–1.07). The field-of-view size was adapted to each individual’s physique. A medium smooth convolution kernel (B30f) was used. A precontrast scan was obtained before administration of 2.0 mL/kg of nonionic contrast material (iopromide, Ultravist 300, Bayer Schering Palmar), followed by a 20-mL saline chaser bolus injected at a fixed injection duration of 30 seconds. Using a bolus-tracking technique, the late arterial phase scan was started 18 seconds after the Hounsfield Units (HU) in the abdominal aorta reached 100 HU. The portal venous phase scan was obtained 35 seconds after the end of the arterial phase scan.

### Color mapping of the liver AEF

The CT data set was transferred to a workstation (Leonardo; Siemens Medical Solutions, Erlangen, Germany), and a quantitative AEF map of the whole liver was created using dedicated software (Hepacare; Siemens Medical Solutions) as previously described [11,15]. Briefly, the three-phase data sets were precisely aligned in three dimensions by non-rigid warping techniques to correct the mismatches in the breath-hold images [8]. During registration, the unenhanced image is used as a reference, and the arterial and portal phase CT images are registered on unenhanced phase CT images. Unenhanced CT images are then subtracted from the arterial and portal venous phase images and AEF percentages, i.e., the ratio of the absolute increment of attenuation on the arterial phase to that of the portal phase multiplied by 100, are mapped on the color map pixel by pixel (color map scale: purple, 0%; red, 100%) [8].

### Image analysis

**Fig 1:**
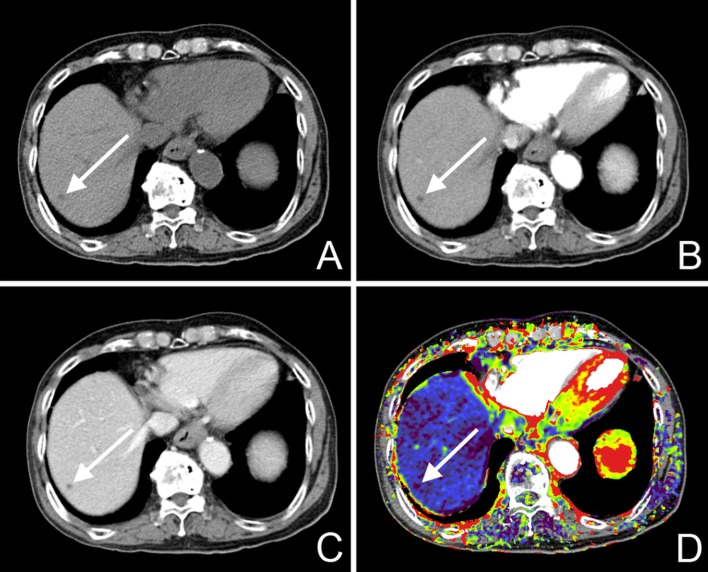
A 64-year-old female patient with sigmoid colon cancer showed a tiny low-attenuating lesion in the S8 of the liver on a dynamic CT scan (A–C). Because this lesion showed no interval change for 2 years on follow-up imaging studies and presented as a high SI lesion on T2-weighted MR images, it was diagnosed as a hepatic cyst. The AEF color map presented the lesion as a nodule (arrow) with decreased AEF (D).

**Fig 2:**
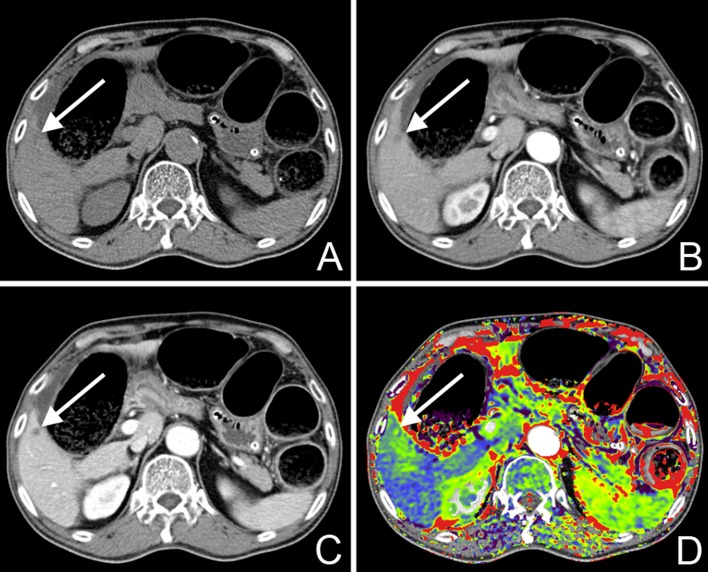
A 68-year-old male patient who was diagnosed with rectosigmoid colon cancer had a low attenuating focal lesion in the S5 of the liver on dynamic CT (A–C). Note that the lesion-adjacent parenchyma also exhibited increased AEF (green) on the AEF color map (D). The lesion was confirmed to be metastatic adenocarcinoma from the histopathologic report.

Two radiologists (C.W.S and K.K.A with 6 and 5 years of experience in abdominal radiology, respectively) independently analyzed the multiphasic CT image sets. To avoid any possible recall bias, they analyzed the combined image sets six weeks later. The first image set consisted of unenhanced, arterial, and portal phase images, and the second image set was a combined image set of the AEF maps of the liver and multiphasic CT images. The observers were aware of the overall goal of the study before the review and knew that the patients had been diagnosed with colorectal cancer. The first and second image sets were reviewed in stack mode using the picture archiving and communication system (PACS). All observers were asked to record the number, size, and segment of the lesions and to grade them by level for the diagnosis of liver metastasis. Size was assessed by measuring the maximum diameter of the lesion on a transverse CT image. The diagnostic confidence for each lesion was scored according to a five-point scale: Score of 1 = definite benign lesion; score of 2 = probably benign lesion; score of 3 = possible metastasis; score of 4 = probably metastasis; and a score of 5 = definite metastasis. Observers were aware that, for statistical analyses, scores of 4–5 would be considered indicative of metastatic lesions and a score of 3 would be considered indicative of a lesion which needs further investigation by liver MRI to differentiate hepatic metastasis from benign hepatic cyst. If there was a mismatch between the CT and AEF color map during evaluation of the second image sets, the lesion was rated as score 3. The diagnostic criteria for hepatic cyst (Fig. 1) and liver metastasis (Fig. 2) by CT and AEF color mapping are summarized in Table 1 [11,16,17].

**Table 1:** CT and AEF color map diagnostic criteria for the assessment of focal hepatic lesions.

	CT	AEF color map
**Hepatic metastasis**	Low- or iso-attenuating masses with rim enhancement	Increased AEF value of the whole lesion or peripheral portion of the lesion, compared with liver parenchyma[11]
	Irregular margin or central necrosis	Increased AEF value of lesion-adjacent parenchyma compared with tumor-free parenchyma
	Incomplete centripetal enhancement pattern	
**Hepatic cyst**	Water attenuation (0–10 HU)	Close to 0% (purple color) or 0% (defect on AEF map)
	No enhancement	No increased AEF value of lesion-adjacent liver parenchyma, compared with lesion-free liver parenchyma
	Imperceptible wall	

CT, computed tomography; AEF, arterial enhancement fraction; HU, Hounsfield unit.

### Quantitative analysis of AEF values

AEF values were quantitated in both benign-lesion-adjacent parenchyma and metastatic-tumor-adjacent parenchyma. Lesion-adjacent liver parenchyma was defined as normal liver parenchyma which contained hepatic metastasis or a cyst in the same segment. Region of interests (ROIs) were drawn manually in each segment without including the lesion itself or major visible vessels. If the patient had both metastasis and hepatic cyst in the same segment, it was considered metastasis-adjacent liver parenchyma.

### Statistical Analysis

All statistical analyses were performed by a medical statistician. Comparison of the mean AEF values of tumor-adjacent and benign-lesion—adjacent parenchyma were carried out by the independent- sample t-test. *P* values less than 0.05 were considered statistically significant. The analysis was performed using SAS software (version 9.2, Cary, NC, USA). For imaging interpretation, ROC analysis was performed using SPSS software (PASW Statistics, version 20; SPSS, Chicago, IL, USA). Interobserver agreement was evaluated using Cohen kappa (k) statistics. A k-value of 0–0.20 indicated slight agreement; 0.21–0.40, fair agreement; 0.41–0.60, moderate agreement; 0.61–0.80, substantial agreement; and 0.81–1.00, almost perfect agreement [18]. Sensitivity, specificity, positive predictive value (PPV), negative predictive value (NPV), and accuracy of diagnostic performance were compared using logistic regression with the generalized estimating equation (GEE) method [19].

## Results

### Patients

Forty-six patients (mean age, 60.8 years ± 11.4 [standard deviation]), 32 men (mean age, 60.3 years ± 10.4) and 14 women (mean age, 62.1 years ± 13.7), were included in the study. There were a total of 123 lesions and, among them, 32 (26.0%) lesions were hepatic metastases and 91 (74.0%) were benign. Overall, 16 (34.8%) patients had hepatic metastasis and 6 (13.0%) of them had both metastasis and hepatic cysts. Among 32 hepatic metastases (11.8 ± 6.6 mm), there were 19 lesions less than 10 mm in diameter (7.2 ± 1.7 mm), and 13 lesions more than 10 mm in diameter (18.5 ± 5.0 mm). In 91 hepatic cysts (6.6 ± 3.8 mm), 84 lesions were less than 10 mm in diameter (5.6 ± 1.6 mm), and 7 lesions more than 10 mm in diameter (18.2 ± 3.8 mm). A final diagnosis was established by histopathology for 22 metastatic lesions in 11 patients and by serial imaging studies for 10 lesions in 5 patients. For 10 metastatic lesions in 5 patients, following imaging studies were done with a combination of MDCT (follow up interval: 27.0 ± 17.0 months) and MRI at 12-month intervals. Cysts were diagnosed based on follow-up MDCT (n = 9), MRI (n = 3), or a combination of MDCT and MRI (n = 24) with a mean follow up interval of 32.3 ± 15.4 months.

### Diagnostic performance of AEF color maps

The mean value of the area under the ROC curve (Az) increased when multiphasic CT images and AEF color maps combined were compared with evaluation based only on multiphasic CT images from 0.698 to 0.897 (reader 1) and from 0.825 to 0.945 (reader 2) (P value < 0.001 and 0.002, respectively). This increase was especially significant for lesions less than 1 cm in diameter, increasing from 0.702 to 0.888 (reader 1) and from 0.768 to 0.958 (reader 2) (P value = 0.001 and 0.001, respectively). The difference in the mean Az value of lesions of more than 1 cm in diameter was not statistically significant (Table 2).

**Table 2:** Area under the receiver-operating characteristic curve of multiphasic CT and AEF color maps for the detection of colorectal liver metastases according to size.

	Reader 1		Reader 2	
	A_Z_ value	*P* value	A_Z_ value	*P* value
**All lesions**		<0.001		0.002
** CT**	0.698 ± 0.0258		0.825 ± 0.0412	
** CT+AEF color map**	0.897 ± 0.2285		0.945 ± 0.0165	
**<1cm**		0.001		0.001
** CT**	0.702 ± 0.0269		0.768 ± 0.0591	
** CT+AEF color map**	0.888 ± 0.0403		0.958 ± 0.0152	
**≥1cm**		0.143		0.071
** CT**	0.643 ± 0.0922		0.714 ± 0.1010	
** CT+AEF color map**	0.786 ± 0.1010		0.786 ± 0.1010	

Az, the area under the receiver-operating characteristic curve; CT, computed tomography; AEF, arterial enhancement fraction.

The diagnostic performance is summarized in Table 3. The accuracy of using combined image sets including AEF color maps was significantly higher compared with using only multiphasic CT for both readers (*P* values ≤ 0.001 and 0.013). This increase in accuracy with the combined image sets was also higher for lesions less than 1 cm in diameter (*P* value = < 0.001 and 0.021 for two readers). However, for lesions exceeding 1 cm in diameter, the accuracy was higher or at least the same when CT was combined with color maps compared with multiphasic CT, but the difference was not statistically significant (*P* values = 0.158 and 0.318 for two readers)

**Table 3:** Diagnostic performance of hepatic metastasis detection on multiphasic CT and AEF color maps.

Reader and Lesion		Reader 1			Reader 2		
		<1cm	≥1cm	Total	<1cm	≥1cm	Total
		(n = 103)	(n = 20)	(n = 123)	(n = 103)	(n = 20)	(n = 123)
**Sensitivity (%)**	**CT**	100	100	100	63.2	100	78.1
	**CT+AEF map**	89.5	100	93.8	100	100	100
	***P* value**	0.158	> 0.999	0.158	0.008	> 0.999	0.008
**Specificity (%)**	**CT**	40.5	28.6	39.6	90.5	42.9	86.8
	**CT+AEF map**	88.1	57.1	85.7	91.6	57.1	89
	***P* value**	<0.001	0.166	<0.001	0.655	0.319	0.414
**Accuracy (%)**	**CT**	51	75	55.3	85.3	85	84.6
	**CT+AEF map**	88.2	85	87.8	93.1	85	91.9
	***P* value**	<0.001	0.158	<0.001	0.021	0.318	0.013
**PPV (%)**	**CT**	27.5	72.2	36.8	60	76.5	67.6
	**CT+AEF map**	63	81.3	69.8	73.1	81.3	76.2
	***P* value**	<0.001	0.158	<0.001	0.13	0.318	0.1
**NPV (%)**	**CT**	100	100	100	91.5	100	91.9
	**CT+AEF map**	97.3	100	97.5	100	100	100
	***P* value**	0.158	>0.999	0.158	0.008	>0.999	0.008

CT, computed tomography; AEF, arterial enhancement fraction.

There were two false-negative lesions in two patients that were not verifiable on the AEF color maps but were verifiable on multiphasic CT. A retrospective review of these lesions revealed that they measured 4 mm and 6 mm in diameter, but their location at the liver dome may have resulted in mismatches in the multiphase images due to patient breathing. There were five false-positive lesions in three patients that had been rated either 1 or 2 by multiphasic CT and were later rated 3 or 4 with the AEF color map. All three patients showed mismatches in the multiphase images due to poor breath holding, and two of the five lesions were located adjacent to the diaphragm.

### Quantitative color mapping of liver AEF

In 46 patients, there were 63 benign-lesion-adjacent parenchyma and 28 metastatic-tumor-adjacent parenchyma. The mean AEF value of metastatic-tumor-adjacent parenchyma (35.1 ± 27.2) was significantly higher than that of benign-lesion-adjacent parenchyma (22.8 ± 19.4) (*P* value = 0.016).

### Interobserver Agreement

For multiphasic CT imaging, the k value for the two observers was 0.274, indicating fair interobserver agreement, but the k value dropped to 0.180 when the lesion was less than 1 cm. When combined with color mapping of the hepatic AEF, the k value for all lesions was 0.766, indicating good agreement, and even for lesion less than 1 cm, the k value was 0.721 (Table 4).

**Table 4:** k values for interobserver agreement.

Observer Comparison	CT	CT+AEF map	*P* value
All	0.274	0.766	<0.001
Size < 1 cm	0.18	0.721	<0.001

CT, computed tomography; AEF, arterial enhancement fraction.

## Discussion

Our results suggest that the diagnostic performance for small hepatic metastases in patients with colorectal cancer, especially for lesions less than 1 cm in size, can be improved by combined review of CT images and AEF color mapping. The interobserver agreement between two readers was also improved after adding AEF color mapping. The AEF value was significantly higher in metastatic-tumor-adjacent parenchyma than in lesion-free liver parenchyma.

For patients with hepatic metastases from colorectal cancer, the prognosis is better with surgical removal or percutaneous ablation of the hepatic metastases [20]. Thus, accurate diagnosis of hepatic metastasis is important. Although CT is the primary diagnostic tool for preoperative evaluation and postoperative surveillance, MRI showed better diagnostic performance in characterization of small hepatic metastases [21,22]. State-of-the-art MDCT uses beam collimation of less than 1 mm, but image reconstruction in these studies involved images with 3 mm or 5 mm collimation. This could have resulted in ill-defined margins or pseudoenhancement and a decrease in diagnostic accuracy and interobserver agreement, especially in lesions less than 1 cm in size. Furthermore, with inherent high tissue contrast and hepatocyte-specific contrast agent or diffusion-weighted images, liver MRI can allow for high diagnostic accuracy (Az value, 0.965) and high sensitivity (97.4%), even with small hepatic metastases [7,13]. Consequently, additional MRI after abdominal CT scan can assist in the characterization of small liver lesions [21], despite being both time-consuming and expensive. In our study, the diagnostic performance of combined sets of quantitative AEF color mapping and multiphasic CT images (Az value, 0.888 and 0.958) was higher than that of CT only (Az value, 0.78) and close to that of liver MRI (Az value, 0.96) [21]. Furthermore, interobserver agreement was also improved after combined review of CT images and AEF color maps. Considering that AEF color maps can be obtained from existing CT images without additional time or expense, they could easily be used in daily clinical practice before performing other diagnostic imaging modalities.

Perfusion imaging of the liver is known to have the potential to improve the early detection of regional liver disease, including hepatic metastasis through the resolution of hepatic arterial and portal venous components [23]. In the presence of metastasis, an increase in hepatic arterial perfusion compared with total hepatic perfusion has been reported [24–26]. However, although perfusion CT could provide additional information for the diagnosis of metastasis, it could not be used in a clinical setting due to the relatively high radiation exposure during examination [23]. When used as a post-processing technique, quantitative measurement of hepatic AEF has the potential to replace perfusion imaging, since the AEF of tumor-adjacent parenchyma is higher than that of lesion-free liver parenchyma without the need for additional radiation exposure [11]. We similarly found that the AEF of tumor-adjacent parenchyma was higher than that of benign-adjacent and lesion-free parenchyma. [4,27]

Although previous studies have not specifically described false-positive and false-negative diagnoses [8,11,27], two false-negative and five false-positive diagnoses were made in our study. The three-dimensional non-rigid warping techniques used by the Hepacare software to align three-phase image data sets might not have been sufficient to correct data mismatch in the liver dome, which is the most vulnerable to diaphragm and heart motion. Hence, application and interpretation of AEF color mapping needs to be carried out with caution in patients who cannot hold their breath well or in patients who have lesions in the liver dome.

Our study had some limitations. First, it was a retrospective study based on analysis of medical records, thus selection bias might exist. Second, the study population was relatively small. Third, only hypovascular metastases were evaluated. Forth, the benign lesions only included hepatic cysts. Other benign lesions such as small hemangioma, hamartoma, or granuloma could also have been present. However, the relatively low incidence of other benign lesions compared with hepatic cysts suggests that our results may be applicable in daily practice. Fifth, as mentioned above, there were two false-negative and five false-positive diagnoses made on the AEF color map, and these misdiagnoses were mainly due to data mismatch caused from motion artifacts. Thus, the added value of a AEF color map can be limited especially for lesions located at the liver dome, which is vulnerable to diaphragm and heart motion. Sixth, only 22 lesions among the total 123 lesions were confirmed through pathologic reports while the final diagnosis of the remaining 121 lesions were made based on 1-year follow up imaging studies. Previous studies with small hepatic metastases showed high diagnostic performances of gadolinium-EOB-DTPA enhanced and diffusion-weighted MRI with Az values ranging from 0.926 to 0.983 but they also reported false positive and negative cases and this might cause wrong diagnoses of standard references. [7,13] Hence, although this might be true for very few cases, there is a possibility of wrong diagnosis on MR. However, this study had a follow up period of at least 12 months and this is thought to have minimized the risk of misdiagnosis. Seventh and last, there was a 6-week interval between the two imaging analysis sessions to avoid recall bias but this method of evaluating the true added value of AEF color mapping might have its own limitations.

In conclusion, AEF color mapping can allow improvement of diagnostic performance for the differential diagnosis of small colorectal hepatic metastases from hepatic cysts, especially in lesions less than 1 cm in diameter, without additional radiation exposure.
